# Characterization of Bioactive Composition and Structural Properties of Epidermis and Cortex Tissues in Edible Cactus [*Opuntia ficus-indica* (L.) Mill.] Cladodes at Different Maturity Stages

**DOI:** 10.3390/foods15142520

**Published:** 2026-07-16

**Authors:** Kriangsuk Boontiang, Tipaukson Chaikwang, Theeraphan Chumroenphat, Sirithon Siriamornpun

**Affiliations:** 1 Department of Agricultural Technology, Mahasarakham University, Kantarawichai, Maha Sarakham 44150, Thailand; kriangsuk.b@msu.ac.th; 2 Research Unit of Thai Food Innovation (TFI), Mahasarakham University, Kantarawichai, Maha Sarakham 44150, Thailand; 3 Department of Food Technology and Nutrition, Faculty of Technology, Mahasarakham University, Kantarawichai, Maha Sarakham 44150, Thailand; 67010853502@msu.ac.th; 4 Cosmetic Science and Spa Program, Faculty of Thai Traditional and Alternative Medicine, Ubon Ratchathani Rajabhat University, Ubonratchathani 34000, Thailand; theeraphan.c@ubru.ac.th

**Keywords:** cacti, phenolics, flavonoids, organic acid, sustainability

## Abstract

*Opuntia ficus-indica* (L.) Mill., an edible cactus, has gained increasing attention as a potential food resource due to its adaptability and nutritional value. This study aimed to characterize the bioactive compound profiles and structural characteristics of the epidermis and cortex of cladodes across different maturity stages. Numerically higher levels of bioactive compounds were generally observed in the epidermis than in the cortex, particularly gentisic acid (384.58–668.23 µg/g DW in the epidermis vs. 191.11–426.69 µg/g DW in the cortex) and protocatechuic acid (154.04–234.45 µg/g DW in the epidermis vs. 40.78–118.44 µg/g DW in the cortex). The epidermis and cortex also exhibited distinct organic acid profiles, with oxalic and malic acids being the predominant organic acids detected in both tissues. FTIR analysis further revealed a more pronounced O–H absorption band associated with water molecules in the cortex than in the epidermis. Furthermore, stage-dependent variations were observed in both bioactive composition and structural characteristics. Organic acids exhibited stage-dependent variations in both tissues. Oxalic, malic, and succinic acids generally occurred at higher levels during the later maturity stages, whereas fumaric acid was more abundant at the earliest maturity stages. Their concentrations ranged from 13.61–47.20, 10.74–16.27, 7.56–14.47, and 0.51–20.46 mg/g DW in the epidermis, and 8.19–46.82, 9.67–52.26, 2.76–14.72, and 0.27–34.41 mg/g DW in the cortex, respectively. These findings provide baseline information on the compositional and structural characteristics of edible cactus cladodes and may support future studies on their potential food-related applications.

## 1. Introduction

At present, global warming has become a major issue affecting people worldwide. Growing levels of greenhouse gas production have led to elevated global temperatures, with the average temperature increasing by about 1.1 ± 1.2 °C [1]. These changes in climate have resulted in more frequent and intense heat waves, which significantly impact human health and productivity, including agricultural production [2]. Among the most consequential domains affected by anthropogenic climate change is agricultural productivity. Empirical projections indicate that continued warming trends carry a substantial risk of precipitating significant reductions in global crop yields, with far-reaching implications for food security at both regional and international scales. Staple crops, notably rice (*Oryza sativa*), wheat (*Triticum aestivum*), maize (*Zea mays*), cassava (*Manihot esculenta*), potatoes (*Solanum tuberosum*), and other vegetable crops, exhibit particular sensitivity to these thermal perturbations, rendering them disproportionately vulnerable to climate-induced yield losses [3,4]. Given that these species collectively constitute the cornerstone of global caloric supply, their susceptibility to warming-associated stress presents a significant threat to worldwide food security [3]. These challenges have increased interest in edible cactus (*Opuntia ficus-indica*) as a climate-resilient crop due to its tolerance to drought and high temperatures. Understanding the tissue-specific composition and structural characteristics of its cladodes is essential for food applications.

*Opuntia ficus-indica* (L.) Mill. is an edible succulent cactus belonging to the family Cactaceae. Native to Mexico, it has been widely cultivated in tropical and subtropical regions, including Latin America, Africa, the Mediterranean region, and the Middle East, owing to its remarkable adaptability to arid and semi-arid environments [5]. Its well-developed root system and fleshy cladodes enable efficient water storage, allowing the plant to withstand high temperatures, elevated carbon dioxide concentrations, and prolonged drought conditions [6]. These adaptive characteristics have contributed to its broad geographical distribution, high genetic diversity, and worldwide cultivation [7]. In addition to its ecological resilience, *O. ficus-indica* cultivation can reduce soil moisture evaporation and enhance windbreak and sand stabilization, highlighting its environmental significance [8]. Consequently, *O. ficus-indica* has gained increasing attention as a sustainable crop with considerable potential to support agricultural production under climate change and other environmental challenges.

In recent years, *O. ficus-indica* has gained increasing attention as a potential food and nutritional resource. The plant is rich in water, dietary fiber, and essential minerals such as potassium, calcium, magnesium, sodium, manganese, iron, and zinc [6,9]. In addition, all parts of the *Opuntia* spp. plant are edible, including the cladodes, fruits, seeds, and flowers [5]. Several studies have evaluated the nutritional value of *O. ficus-indica*. Extracts from its flowers have been reported to contain high levels of bioactive compounds particularly polyphenols and flavonoids such as gallic acid, isorhamnetin 3-O-rutinoside, and isorhamnetin 3-O-galactoside. These compounds exhibit strong antioxidant activity and show potential for use as natural preservatives in food systems [9,10]. Furthermore, cactus consumption has been associated with various health benefits, including the prevention of diabetes [11] and hypercholesterolemia [12]. Young cladodes at the mature growth stage are often reported to contain higher levels of soluble dietary fiber, protein, and bioactive compounds such as polyphenols, contributing to strong antioxidant, anti-inflammatory, and cell-protective effects [13,14]. In contrast, older cladodes tend to accumulate higher levels of insoluble dietary fiber and minerals, especially calcium, while the levels of some bioactive compounds decrease [15]. The interest in *O. ficus-indica* is not only due to its ability to grow under harsh environmental conditions but also its promising nutritional value [16]. Previous studies have demonstrated that cactus age significantly influences both the quantity and composition of bioactive compounds. Chaicharoenaudomrung et al. [17] investigated Golden barrel cactus (*Echinocactus grusonii*) and reported that 3-year-old cacti contained higher levels of bioactive compounds, particularly phenolics, lutein, and chlorophyll derivatives chlorophyll a, b, pheophytin a, and b, along with greater antioxidant activity, compared to 6-year-old plants, which showed a decline in these compounds. This suggests that younger cacti may possess greater bioactive potential. In addition, Farias et al. [18], who examined the maturity stages of cladodes in *O. ficus-indica* cv. *Gigante* and cv. *Redonda*, and found that bioactive compounds varied with maturity stages. Phenolic content was higher at early stages, whereas chlorophylls, carotenoids, and flavonoids were more abundant at intermediate to late stages. Moreover, ascorbic acid content increased with advancing maturity. These findings highlight that both age and maturity stages are key factors influencing the bioactive composition of cacti. However, existing studies have primarily focused on overall nutritional composition and biological properties, whereas comparative analyses of the structural characteristics of specific cladode tissues, particularly the epidermis and cortex across different growth stages, remain limited.

Therefore, this study aimed to characterize the bioactive compound profiles and structural characteristics of the epidermis and cortex of *O. ficus-indica* (L.) Mill. cladodes across different maturity stages. The study provides baseline information on the compositional and structural characteristics of edible cactus cladodes and contributes to a better understanding of their variation during development. These findings may support future studies evaluating the biological characteristics, functional properties, and potential utilization of cactus cladodes as a locally available raw material for food-related applications.

## 2. Materials and Methods

### 2.1. Materials and Reagents

**Figure 1:**
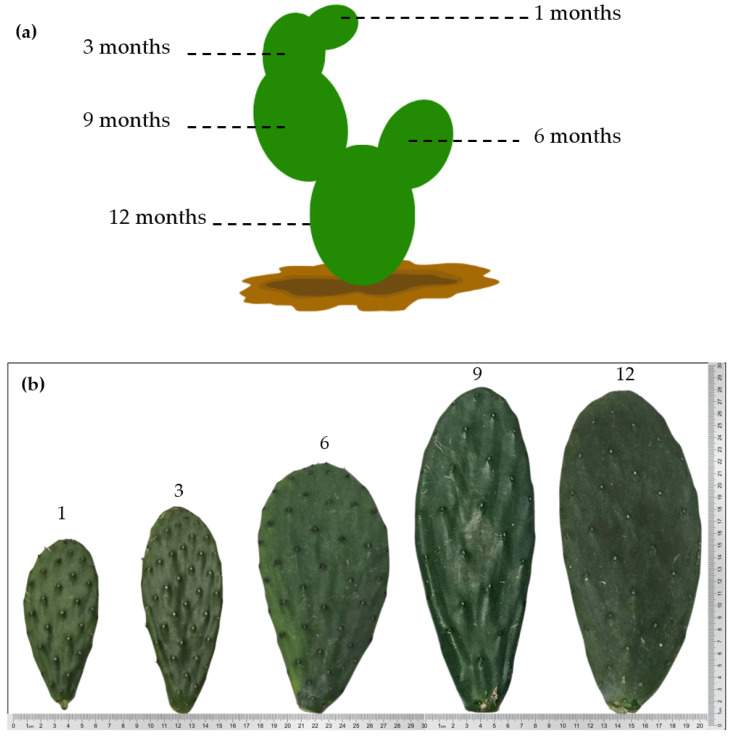
Developmental stages of *O. ficus-indica* (L.) Mill. cladodes. (**a**) Schematic illustrations and (**b**) representative photographs showing cladodes harvested at 1, 3, 6, 9, and 12 months after planting.

Cladodes of *Opuntia ficus-indica* (L.) Mill. at five developmental stages (1, 3, 6, 9, and 12 months), as shown in Figure 1, were collected between September and December 2025 from the MSU Cactus Nursery, Department of Agricultural Technology, Faculty of Technology, Mahasarakham University, Maha Sarakham, Thailand. All plants were cultivated under identical environmental conditions, and cladode age was determined from the cultivation records maintained by the nursery. For each developmental stage, 30 uniformly sized cladodes were harvested from multiple healthy plants free from visible symptoms of pests or diseases. Three cladodes were randomly selected from the collected material and used to prepare a representative composite sample. The epidermis and cortex were manually separated from each of the three selected cladodes prior to drying. The epidermal tissues from the three cladodes were pooled to obtain one composite epidermis sample, while the cortical tissues were pooled separately to obtain one composite cortex sample for each developmental stage. Each composite sample was dried, ground into a fine powder, thoroughly homogenized, and stored under appropriate conditions until analysis. All chemicals were obtained from Sigma-Aldrich Co. (St. Louis, MO, USA). Analytical- or HPLC-grade standards (≥90% purity) were used for the determination of bioactive compounds, antioxidant activity, phenolic acids, flavonoids, and organic acids.

### 2.2. Cladode Sample Preparation

**Figure 2:**
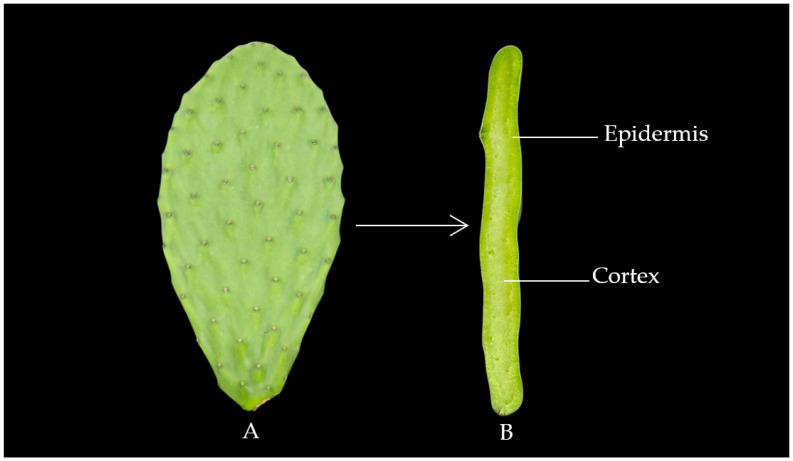
Images of edible cactus (*O. ficus-indica* (L.) Mill.) cladodes: (**A**) intact cladode; (**B**) cross-section.

Cladodes at five maturity stages (1, 3, 6, 9, and 12 months) were thoroughly washed with distilled water. The samples were then sectioned and separated into two tissue parts, as illustrated in Figure 2 and described in Table 1. The epidermis and cortex were manually separated following the approach reported by Osorio-Esquivel et al. and Fernández-Martínez et al. [19,20], with slight modifications. All tissue separations were performed by the same operator. The outer tissue (designated as the epidermis) was carefully peeled manually using a sharp knife based on the visible green outer layer while avoiding excessive removal of the underlying tissue. The remaining inner tissue was designated as the cortex. Because cladodes at different maturity stages varied in size and thickness, the thickness of the removed outer layer was not standardized and may have varied among samples. Therefore, the terms “epidermis” and “cortex” are used descriptively in this study rather than as strict anatomical classifications. Throughout the separation process, careful manual handling was applied to minimize cross-contamination between the epidermis and cortex. The relative mass of the separated tissues was not recorded. The separated tissues were freeze-dried, ground into a fine powder, passed through a 50-mesh sieve, and stored at −20 °C until further analysis.

### 2.3. Cladode Extraction for Bioactive Compounds and Antioxidant Activity

The extraction of cactus tissues was performed following a modified method of Boonarsa et al. [21]. A 5 g portion of each homogenized composite sample was extracted with 25 mL of 80% ethanol and incubated in a shaker incubator at room temperature for 18 h. The extracts were collected and stored in amber bottles until further analysis of bioactive compounds. Each extract was measured in triplicate, and the reported values represent the mean of the three determinations.

### 2.4. Total Phenolic Content (TPC) Analysis

TPC was determined according to the method described by Chaikwang et al. [22]. A 0.2 mL sample was mixed with 0.5 mL of 10% Folin–Ciocalteu reagent and thoroughly vortexed to ensure homogeneity. Subsequently, 2.25 mL of 7% Na_2_CO_3_ solution was added. The reaction was allowed to proceed for 90 min in the dark at room temperature. The absorbance of the samples was determined at 725 nm using a UV–visible spectrophotometer (UV-1700, Shimadzu, Tokyo, Japan). A calibration curve was prepared using gallic acid standard solutions. TPC was expressed as mg gallic acid equivalents (GAE)/100 g DW.

### 2.5. Total Flavonoid Content (TFC) Analysis

TFC was determined according to the method of Chumroenphat and Saensouk [23]. A 500 µL portion of the extract was mixed with 2.25 mL distilled water, followed by the addition of 150 µL of 5% NaNO_2_ and 300 µL of 10% AlCl_3_·6H_2_O. After a reaction period of 6 min, 1 M NaOH 0.1 mL was introduced. Measurement of absorbance was carried out at 510 nm. A calibration curve was prepared using quercetin standard solutions. TFC was expressed as mg quercetin equivalents (QE)/100 g DW.

### 2.6. DPPH Free Radical Scavenging Assay (DPPH) Analysis

The DPPH was determined according to the method described by Siriamornpun et al. [24] with slight modifications. The extract 0.5 mL was reacted with 4.5 mL of 0.06 mM DPPH solution, mixed, and kept in the dark at room temperature for 30 min. Measurement of absorbance was carried out at 517 nm. A calibration curve was prepared using L-ascorbic acid standard solutions. The DPPH radical scavenging activity was expressed as mg L-ascorbic acid/100 g DW.

### 2.7. Analysis of Antioxidant Activity by FRAP Assay

The FRAP of the samples was assessed following the procedure described by Kessara Mungkunkoth et al. [25], with slight modifications. An aliquot of 0.06 mL of the extract was mixed with 0.18 mL of distilled water and 1.8 mL of FRAP reagent, consisting of acetate buffer, TPTZ, and FeCl_3_ in a 10:1:1 ratio. The mixture was vortexed and incubated at 37 °C for 4 min. Absorbance was measured at 593 nm. A calibration curve was prepared using FeSO_4_ standard solutions. The ferric reducing antioxidant power (FRAP) was expressed as mg FeSO_4_ equivalents/100 g DW.

### 2.8. HPLC Analysis of Phenolic Acids and Flavonoids

Phenolic acid and flavonoid contents were determined following the method described by [26] with slight modifications. A 1 g portion of each sample was extracted with 20 mL of water/methanol (80:20, *v*/*v*) and shaken at 37 °C and 150 rpm for 12 h. The extracts were filtered through a 0.22 μm nylon membrane filter prior to HPLC analysis. Phenolic acids and flavonoids were analyzed using an HPLC system (Series 20, Shimadzu, Kyoto, Japan) equipped with a C18 column (4.6 mm × 250 mm, 5 μm; GL Sciences Inc., Tokyo, Japan). The mobile phase consisted of 1% acetic acid in water (solvent A) and acetonitrile (solvent B). Gradient elution was performed as follows: 0–5 min, 5–9% B; 5–15 min, 9% B; 15–22 min, 9–11% B; 22–38 min, 11–18% B; 38–43 min, 18–23% B; 43–44 min, 23–90% B; 44–45 min, 90–80% B; 45–55 min, 80% B; and 55–60 min, 80–5% B, followed by a 5 min re-equilibration at 5% B before the next injection. The flow rate was 0.8 mL/min, the column temperature was maintained at 38 °C, and the injection volume was 20 μL. Detection was performed using a photodiode array detector at 280 nm for hydroxybenzoic acids, 320 nm for hydroxycinnamic acids, and 370 nm for flavonoids. Individual compounds were identified by comparing their retention times and UV spectra with those of authentic standards. Quantification was performed using external calibration curves prepared from authentic standards. Each extract was injected into the HPLC system in triplicate, and the reported values represent the mean of three technical (instrumental) injections. Calibration curves were established using five concentration levels (6.25–100 μg/mL) for each analyte. Linearity was evaluated by linear regression analysis, and the limits of detection (LOD) and limits of quantification (LOQ) were automatically calculated using Shimadzu LabSolutions software ver. 5.82 SP1 (Shimadzu Corporation, Kyoto, Japan) based on the standard deviation of the response and the slope of the calibration curve. Method accuracy was evaluated by recovery experiments using cactus samples spiked with known concentrations of authentic standards prior to extraction and HPLC analysis. Detailed validation parameters, including calibration ranges, regression equations, coefficients of determination (R^2^), LOD, LOQ, and recovery values, are presented in Supplementary Table S1.

### 2.9. Organic Acids Determination

Organic acid analysis was performed according to the method described by [27]. Samples were analyzed using an HPLC system (20 Series, Shimadzu, Kyoto, Japan) under isocratic conditions with 0.05 M sulfuric acid as the mobile phase at a flow rate of 0.5 mL/min. Separation was performed on an Aminex^®^ HPX-87H column (300 mm × 7.8 mm, 5.0 µm; Bio-Rad Laboratories, Hercules, CA, USA) maintained at 80 °C, with an injection volume of 25 µL. Detection was carried out using a UV–visible detector at 210 nm. Organic acids were identified by comparing their retention times with those of authentic standards (oxalic, malic, succinic, and fumaric acids) and quantified using external calibration curves prepared from the corresponding authentic standards according to the calibration procedure described by Papayrata et al. [28]. Calibration curves were established using five concentration levels (6.25–100 µg/mL), and excellent linearity was obtained for all analytes (R^2^ = 0.9995–0.9998). Detailed calibration parameters are provided in Supplementary Table S2. Each analysis was performed in triplicate, and the results were expressed as mg/g DW.

### 2.10. FT-IR Analysis

FTIR spectra were acquired using an INVENIO S/LUMOS II FT-IR spectrometer (Bruker, Fällanden, Switzerland) equipped with an attenuated total reflectance (ATR) accessory, following the method described by Escárcega Olivares et al. [29] with slight modifications. Approximately 1 mg of sample was placed directly onto the ATR crystal without KBr pellet preparation. Spectra were recorded over the wavenumber range of 4000–400 cm^−1^ at a resolution of 4 cm^−1^ under atmospheric conditions. Baseline correction was applied to all spectra prior to analysis. The resulting ATR-FTIR spectra were analyzed to identify the functional groups present in the samples.

### 2.11. X-Ray Diffraction (XRD)

The diffraction patterns of the cladode samples were collected using a D8 Advance X-ray diffractometer (Bruker, Karlsruhe, Germany) operating at 40 kV and 40 mA. The patterns were recorded over a 2θ range of 5–40° at a scanning rate of 2°/min, according to Ouhammou et al. [30].

### 2.12. Statistical Analysis

Results are presented as mean ± standard deviation (SD). Data normality and homogeneity of variance were evaluated using the Shapiro–Wilk and Levene’s tests, respectively, prior to statistical analysis. One-way analysis of variance (ANOVA), followed by Duncan’s multiple range test (*p* < 0.05), was performed separately for the epidermis and cortex to evaluate differences among developmental stages. The statistical analysis was intended to compare developmental-stage variation within each tissue under the experimental conditions of this study and was not used to evaluate statistical differences between tissue types. Statistical analyses were performed using IBM SPSS Statistics version 17 (IBM Corp., Armonk, NY, USA). Heatmap visualization and Pearson’s correlation analysis were conducted using MetaboAnalyst 6.0 [31], and graphs were prepared using Origin 2022 (OriginLab Corporation, Northampton, MA, USA).

## 3. Results and Discussion

### 3.1. Bioactive Compounds and Antioxidant Activity

Bioactive compounds are associated with nutritional and antioxidant properties. However, their biological relevance depends on factors such as bioavailability and physiological context [32]. The epidermis and cortex of cladodes at different maturity stages are presented in Table 2 and Table 3. The results showed that TPC, TFC, DPPH, and FRAP values varied across maturity stages, with distinct patterns observed between the epidermis and cortex. Instead of following consistent increasing or decreasing trends, these parameters exhibited stage-dependent variations. Specifically, Total phenolic content (TPC) ranged from 164.9 to 407.8 mg GAE/100 g DW, with the highest values observed in the epidermis at 6 months and the cortex at 3 months. Total flavonoid content (TFC) ranged from 402.9 to 918.1 mg QE/100 g DW, with the highest values observed in both the epidermis and cortex at 1 month. DPPH values ranged from 0.3 to 0.9 mg ascorbic acid/100 g DW, with the highest activity observed in the epidermis at 1 month and the cortex at 3 and 9 months. Similarly, FRAP values ranged from 580.4 to 1290.2 mg FeSO_4_/100 g DW, with the highest values observed in the epidermis at 12 months and the cortex at 1 month. Compared with the study by Pensamiento-Niño et al. [33] on cactus flowers, both the epidermis and cortex of cladodes in this study exhibited higher levels of bioactive compounds.

The cladode epidermis generally showed higher numerical values for bioactive compounds and antioxidant activity than the cortex across the evaluated maturity stages. A similar trend was reported by Fundo et al. [34], who observed higher concentrations of bioactive compounds in the rind than in the flesh of cantaloupe. The relatively higher levels of bioactive compounds in the epidermis may be associated with environmental factors that influence the synthesis and accumulation of plant metabolites [35]. As the outermost tissue, the epidermis is directly exposed to environmental conditions, which may contribute to the accumulation of phenolic compounds and other antioxidant-related metabolites through protective secondary metabolic responses [36]. In addition, the observed differences could partly reflect structural differences between the epidermis and cortex that may have influenced the extraction efficiency of bioactive compounds. However, these proposed mechanisms are hypothetical and were not directly investigated in the present study.

Moreover, the levels of bioactive compounds exhibited stage-dependent variations in both tissues. In the epidermis, TPC, TFC, DPPH, and FRAP fluctuated across maturity stages without a consistent increasing or decreasing pattern. Likewise, the cortex also exhibited stage-dependent variations, although the patterns of change differed from those observed in the epidermis. Similar variations in bioactive compounds across developmental stages have also been reported by Farias et al. [18], who studied prickly pear cladodes across four maturity stages and found fluctuations in bioactive compound levels. These variations may be associated with changes in secondary metabolism during plant development, as well as the plant’s physiological responses to environmental conditions [37,38,39]. Overall, variation in the levels of bioactive compounds and antioxidant activity was observed across maturity stages within each cladode tissue, while numerical differences were also observed between the epidermis and cortex. However, the epidermis and cortex differ substantially in their structural characteristics, particularly in the proportion of fibrous and crystalline cell wall components. Although identical extraction conditions were applied to both tissues, these structural differences may have influenced extraction efficiency and, consequently, the measured concentrations of bioactive compounds [40,41]. Further studies on bioavailability, biological activity, functional properties, tissue-specific extraction efficiency, and processing behavior are needed to further evaluate their potential applications as food ingredients.

### 3.2. Phenolic Acid of Cladodes

Phenolic acids are a class of polyphenolic compounds widely distributed in plants, where they play important roles in plant growth and development. They have also been associated with antioxidant properties and potential nutritional benefits, although their biological effects depend on bioavailability and physiological conditions [42]. Different parts of the cladodes, specifically the epidermis and cortex, as well as their maturity stages, influenced the levels of phenolic acids detected, as presented in Table 4 and Table 5. The major phenolic acids identified in both the epidermis and cortex of the cladodes included gentisic acid (191.1–668.2 µg/g DW), with the highest levels observed in the epidermis at 1 month and the cortex at 3 months. Protocatechuic acid (110.7–234.5 µg/g DW) showed the highest concentrations in both tissues at 1 month. Chlorogenic acid (10.9–224.1 µg/g DW) reached peak concentrations in the epidermis and cortex at 1 month. Gallic acid (45.5–57.9 µg/g DW) was also highest in both epidermis and cortex at 1 month. In addition, other important phenolic acids were also detected, including caffeic acid, cinnamic acid, ferulic acid, p-coumaric acid, p-hydroxybenzoic acid, sinapic acid, syringic acid, and vanillic acid. The phenolic acid profile obtained in this study is in agreement with the findings of Godínez-Santillán et al. [43], who reported a similar phenolic composition in Berrycactus (*Myrtillocactus geometrizans*).

Numerically higher total phenolic acid contents were observed in the cladode epidermis than in the cortex. This trend may be associated with the protective function of the epidermis as the outermost tissue of the cladode, where phenolic compounds are generally considered important components of the plant defense system [35,44,45]. In addition, different maturity stages also influenced the levels of phenolic acids. Both the epidermis and cortex exhibited variations in phenolic acid content, with increasing and decreasing trends depending on the stage of growth. This is consistent with the findings of Farias et al. [18], who reported similar variations in cactus across different stages of development. These differences may be attributed to changes in metabolic activity and the plant’s response to environmental stress at each maturity stage [39]. Previous studies have reported that young tissues tend to exhibit higher activity of phenylalanine ammonia-lyase (PAL), a key enzyme involved in phenolic biosynthesis [17]. Phenolic compound levels may further be affected by environmental factors related to plant survival. Therefore, the variation in phenolic acid content observed throughout the maturity stages in both the epidermis and cortex may result from differences in biosynthesis associated with plant growth and adaptation to environmental conditions.

Overall, distinct phenolic acid profiles were observed across maturity stages within the epidermis and cortex. These findings provide baseline information on the antioxidant composition of cladodes, which may support future studies evaluating their suitability for food-related applications.

### 3.3. Flavonoid Content of Cladodes

Flavonoids are important bioactive compounds in plants, playing key roles in growth regulation, defense mechanisms, and responses to environmental stimuli [45]. Different parts of the cladodes, namely the epidermis and cortex, as well as their maturity stages, influenced the flavonoid content detected, as presented in Table 4 and Table 5. Both the epidermis and cortex contained five major flavonoids, namely myricetin, rutin, quercetin, apigenin, and kaempferol. Among these, rutin (49.2–264.2 µg/g DW) and myricetin (40.3–129.4 µg/g DW) were the most abundant. The highest rutin content was observed in the epidermis at 12 months, while the cortex at 1 month exhibited the highest level of myricetin. These findings are consistent with previous reports describing flavonoid profiles in cacti. Cha et al. [46] investigated *Opuntia humifusa* and reported a comparable profile. In addition, the flavonoid composition observed in this study appears to be characteristic of cactus species, in line with the work of Conte et al., who examined the biosynthesis of bioactive compounds across various cactus species [47].

A trend toward higher total flavonoid contents was observed in the cladode epidermis than in the cortex. This observation is consistent with the findings reported by Morales et al. [48], who observed higher levels of bioactive compounds in the outer peel than in the inner tissues of xoconostle, also known as the sour cactus pear (*Opuntia joconostle*).

This difference may be explained by the fact that the epidermis is directly exposed to the external environment, which can stimulate flavonoid synthesis as part of the plant’s defense mechanisms [44]. In addition, cells in the outer peel generally have thicker cell walls and larger vacuoles, which serve as storage sites for flavonoids, including anthocyanins, quercetin, and myricetin, leading to greater accumulation compared to the inner tissues [49]. Different maturity stages also affected flavonoid content. The levels of flavonoids showed both increasing and decreasing trends depending on the stage of growth. This variation aligns with the results reported by Farias et al. [18], who reported changes in total flavonoid content in cactus across different maturity stages. These changes may be related to the dynamic biosynthesis of secondary metabolites that support plant defense and adaptation to environmental conditions throughout development [37,39]. Overall, variation in flavonoid content was observed across maturity stages within both the epidermis and cortex, while numerical differences were also observed between the two tissues. The flavonoid profiles also varied among maturity stages, indicating changes in flavonoid composition during cladode development. These findings provide baseline information on the flavonoid composition of cladodes and may support future studies on their biological characteristics and potential applications.

### 3.4. Organic Acid Content of Cladodes

Organic acids are commonly found in plants and fruits and play an important role in determining flavor and sensory quality [50]. The organic acids identified in the cladodes are presented in Table 6 and Table 7. Differences in cladode tissues, including the epidermis and cortex, as well as maturity stages, influenced the types and levels of organic acids detected. Both tissues contained oxalic, malic, succinic, and fumaric acids. Oxalic, malic, and succinic acids were generally present at higher levels in the later maturity stages, with the highest values observed at 12 months, whereas fumaric acid showed the highest level at 1 month. These acids are commonly reported in plant and fruit tissues, which is consistent with previous studies on cactus fruits [48,51]. The differences in organic acid composition between the epidermis and cortex may be related to variations in tissue structure, while stage-dependent fluctuations likely reflect changes in metabolic activity during development. In both tissues, oxalic, malic, and succinic acid contents were generally higher at later maturity stages, although stage-dependent fluctuations were observed. In contrast, fumaric acid was more abundant at the earlier maturity stages. These variations likely indicate differences in metabolic processes and physiological functions characteristic of *Opuntia* species [52,53]. Organic acids such as citric acid, malic acid, and quinic acid are key intermediates in the Krebs cycle, contributing to energy production and serving as precursors for the synthesis of other biomolecules [48]. Therefore, the observed stage-dependent variations in organic acid levels may reflect adaptive metabolic responses in cactus tissues [47]. Overall, the composition and relative abundance of organic acids varied across maturity stages within both the epidermis and cortex, with numerical differences also observed between the two tissues. These findings provide baseline information on the organic acid composition of cladodes, which may serve as a foundation for future studies investigating their functional properties and potential food-related applications.

### 3.5. Heatmap Visualization and Pearson Correlation Analysis of Bioactive Compounds and Antioxidant Activity

**Figure 3:**
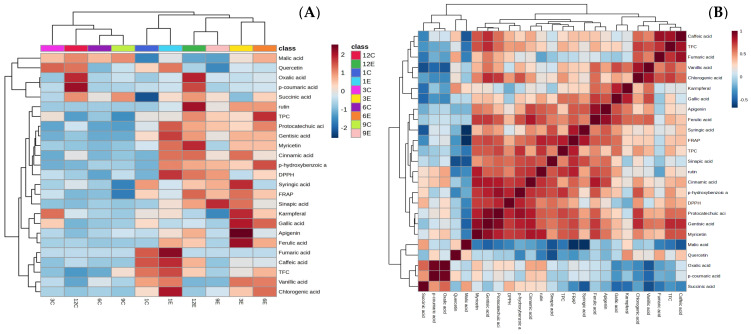
Integrated multivariate analysis of bioactive compounds and antioxidant activity in cactus cladodes at different maturity stages. (**A**) Hierarchical clustering heatmap showing the relative abundance of phenolic acids, flavonoids, organic acids, total phenolic content (TPC), total flavonoid content (TFC), and antioxidant activities (DPPH and FRAP) in epidermis and cortex tissues. Data were normalized using Z-score transformation. Red and blue colors indicate relatively high and low metabolite abundance, respectively. (**B**) Pearson correlation matrix illustrating the relationships among bioactive compounds and antioxidant parameters. Positive and negative correlations are represented by red and blue colors, respectively, with color intensity corresponding to the strength of the correlation.

Hierarchical clustering heatmap and Pearson correlation analyses were performed to comprehensively evaluate the relationships among bioactive compounds, antioxidant activities, maturity stages, and cladode tissues (Figure 3). Together, these multivariate approaches reveal metabolite distribution patterns and their contributions to antioxidant capacity. The hierarchical clustering heatmap (Figure 3A) revealed distinct grouping of samples according to both tissue type and maturity stages, indicating clear variation in phytochemical composition among tissues and maturity stages. Two major clusters were identified, with most epidermis samples grouped separately from cortex samples, reflecting their distinct metabolic profiles. Overall, the epidermis exhibited relatively higher abundances (red color intensity) of total phenolic content (TPC), total flavonoid content (TFC), antioxidant activities (DPPH and FRAP), and several phenolic compounds, including chlorogenic acid, gentisic acid, protocatechuic acid, rutin, myricetin, ferulic acid, and caffeic acid. In contrast, cortex samples were generally characterized by lower relative levels of these metabolites (blue color intensity), although mature cortex tissues exhibited comparatively higher concentrations of certain organic acids, particularly malic and succinic acids. These observations agree with the quantitative data presented in Table 2, Table 3, Table 4, Table 5, Table 6 and Table 7 and support previous studies reporting greater accumulation of phenolic compounds in the outer tissues of plants, where they function as protective metabolites against environmental stresses such as ultraviolet radiation, temperature fluctuations, and oxidative damage [35,44,45]. Hierarchical clustering of the measured variables further demonstrated that antioxidant indices (TPC, TFC, DPPH, and FRAP) were closely associated with several phenolic acids and flavonoids, including chlorogenic acid, gentisic acid, protocatechuic acid, rutin, and myricetin. In contrast, most organic acids formed a separate cluster, suggesting that phenolic metabolites contribute more substantially to antioxidant capacity than primary metabolites. This clustering pattern reflects the well-established role of phenolic compounds as efficient electron and hydrogen donors that enhance radical scavenging and reducing activities.

The Pearson correlation analysis (Figure 3B) further supported the clustering results by demonstrating strong positive associations between antioxidant parameters and phenolic metabolites. TPC, TFC, DPPH, and FRAP exhibited positive correlations with major phenolic acids and flavonoids, indicating that these compounds are closely associated with the antioxidant potential to the antioxidant potential of cactus cladodes. Positive correlations were also observed among structurally and biosynthetically related phenolic acids, including chlorogenic, caffeic, ferulic, and *p*-coumaric acids, as well as among flavonoids such as rutin, quercetin, kaempferol, and myricetin. These relationships are consistent with their common origin within the phenylpropanoid biosynthetic pathway and may reflect coordinated accumulation during cladode development. Conversely, organic acids showed comparatively weaker correlations with antioxidant indices, indicating that although they are essential components of primary metabolism and influence sensory attributes, their contribution to antioxidant activity is less pronounced than that of phenolic compounds.

Overall, the combined heatmap and Pearson correlation analyses revealed distinct metabolite distribution patterns among tissues and maturity stages. The epidermis, particularly at the early and mature maturity stages, generally exhibited higher relative abundances of phenolic compounds and higher antioxidant capacity than the cortex. Furthermore, the close association between antioxidant indices and phenolic metabolites highlights phenolic acids and flavonoids as the major determinants of the antioxidant properties of cactus cladodes. These findings provide baseline information for future studies evaluating the potential utilization of the epidermis as a source of phenolic compounds for food-related applications.

### 3.6. FTIR

**Figure 4:**
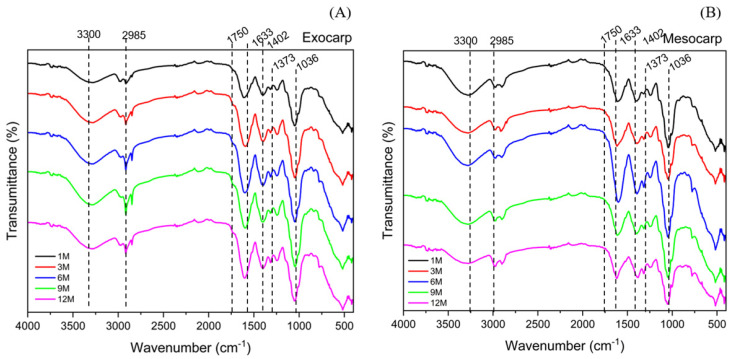
FTIR spectra of epidermis (**A**) and cortex (**B**) tissues from cladodes of *Opuntia ficus-indica* (L.) Mill. at different maturity stages.

FTIR analysis identified the functional groups, as shown in Figure 4. Different parts of the cladode, including the epidermis and cortex, as well as different maturity stages, influenced the functional groups detected in the spectra. An absorption band around 3300 cm^−1^ was observed, attributed to O–H stretching vibrations characteristic of carbohydrates and water retained within the plant cell wall. This observation aligns with previous reports indicating absorption in this region for succulent plants [54,55]. The band observed at approximately 2985 cm^−1^ has been assigned to C–H stretching vibrations associated with pyranose and furanose ring structures, which have been reported for monosaccharides such as galactose, arabinose, and xylose. These monosaccharides are commonly found in the mucilage of plants in the *Opuntia* spp. [56]. In addition, the absorption bands observed at 1750 and 1633 cm^−1^ may be attributed to the vibrations of aldehyde and carboxyl functional groups, which have been previously associated with hemicellulose-containing plant cell walls, including cactus tissues [57]. The bands at 1373, 1243, and 1036 cm^−1^ have been assigned to vibrations of pyranose ring structures that are commonly found in plant polysaccharides [58]. Furthermore, the absorption bands at 1373 and 1430 cm^−1^ observed in this study are consistent with previous reports that have associated these bands with amorphous regions of cellulose [59], including the study by Medina et al. [60] on Colombian cactus species. Nevertheless, this assignment is based on published FTIR interpretations and should be regarded as tentative, since FTIR analysis alone cannot unequivocally confirm cellulose organization or structural modifications. A distinct absorption band at approximately 1402 cm^−1^ has been attributed to the stretching vibration of carboxyl groups in D-galactopyranosyluronic acid, a structural unit commonly found in pectin [55]. Collectively, the FTIR spectra are consistent with the presence of functional groups commonly associated with plant cell wall polysaccharides in cactus cladodes, although FTIR analysis alone cannot unequivocally identify individual polysaccharides.

The FTIR spectra of the epidermis and cortex exhibited similar absorption regions. However, the epidermis showed a stronger absorption band at approximately 2985 cm^−1^, which may be associated with polysaccharide-related structures reported in plant mucilage containing galactose, arabinose, and xylose. These monosaccharides are commonly reported as components of plant cell wall polysaccharides and the outer structural matrix [47]. This interpretation is consistent with the findings of Gheribi et al. [61], who reported that mucilage extracted from the peel of *Opuntia ficus-indica* contains galactose, arabinose, and xylose. In contrast, the cortex exhibited higher absorption in the regions of 3600–3000 and 1040 cm^−1^, which may be associated with O–H stretching vibrations of water molecules and polysaccharide components. This observation may also reflect the relatively higher moisture content typically found in parenchyma tissue than in outer tissues [49]. Variations in the FTIR absorption patterns were also observed across maturity stages, particularly at 1750, 1633, 1642, 1161, and 1040 cm^−1^. These variations may reflect changes in the structural characteristics of cladode polysaccharides [58] and may be associated with O–H groups from cell wall-bound water as well as structural polysaccharides, including hemicellulose- and cellulose-rich components, during plant development. This variability may be attributed to the structural and compositional changes that occur throughout plant growth [15]. However, some studies have reported that maturity stages do not significantly affect the FTIR profile of cactus [62]. Overall, variations in the FTIR spectral characteristics were observed across maturity stages within both the epidermis and cortex, while distinct spectral features were also observed between the two tissues. These variations may reflect differences in tissue composition and organization during cladode development.

### 3.7. XRD

**Figure 5:**
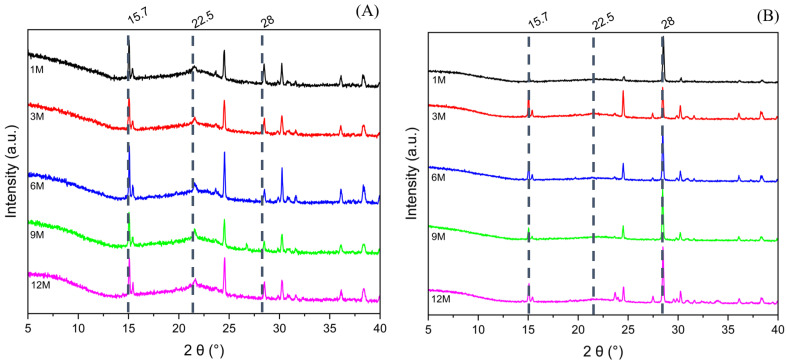
XRD spectra of epidermis (**A**) and cortex (**B**) tissues from cladodes of *Opuntia ficus-indica* (L.) Mill. at different maturity stages.

XRD analysis was used to identify the crystalline structure of compounds present in the cactus samples, as shown in Figure 5. The diffraction profiles of different parts of the cladode, including the epidermis and cortex, as well as samples from different maturity stages, were compared. The results showed similar diffraction patterns in both parts of the cladode. In the epidermis, distinct diffraction peaks were observed at approximately 2θ ≈ 15°, 22.6°, 28°, and 30°. The peaks around 15.7° and 22.6° have been previously reported as characteristic reflections associated with cellulose I and are consistent with observations reported by Cheikh Rouhou et al. [63] for *Opuntia ficus-indica*. The relatively stronger intensity of these peaks in the epidermis may suggest a higher contribution of ordered fibrous components in the outer tissue, which are commonly associated with cellulose-rich cell wall structures together with other structural polysaccharides [64].

Since quantitative crystallinity analysis was not performed in the present study, the XRD results were interpreted qualitatively by comparing the observed diffraction patterns with those reported previously for *O. ficus-indica*. Previous studies have reported relatively high cellulose crystallinity in cactus-derived materials. For example, Maceda et al. [59] reported crystalline cellulose contents of up to approximately 77% in O. ficus-indica, while Habibi et al. [65] observed that outer tissues tended to exhibit higher cellulose crystallinity than inner tissues. However, the reported crystallinity values for O. ficus-indica vary considerably among studies and may be influenced by factors such as tissue type, maturity stages, and cultivation environment [66]. Therefore, the present XRD analysis focused on comparing the diffraction patterns of the epidermis and cortex, and the observed peak characteristics were interpreted qualitatively. The cortex exhibited diffraction peaks at similar positions (2θ ≈ 15°, 22.6°, 28°, and 30°) to those observed in the epidermis, but with noticeably lower peak intensities. This observation may reflect differences in the organization or relative abundance of crystalline cell wall components between the two tissues. In addition, the cortex exhibited a relatively stronger peak at around 2θ ≈ 28° than the epidermis. This peak may be associated with mineral components that have been reported to accumulate in cactus tissues, particularly calcium-containing compounds [67,68]. In addition, comparisons across different maturity stages showed variations in the intensity of diffraction peaks that may be associated with cellulose-rich structures (2θ ≈ 22.5°) and amorphous polysaccharide components, including hemicellulose and lignin (2θ ≈ 15.7°), based on previous reports [30,69]. These variations may reflect structural changes occurring during plant development. Similar variations in crystallinity of rice bran dietary fiber across maturity stages have been reported [22]. Previous studies have associated diffraction features near 2θ ≈ 15° with amorphous polysaccharide components, although the present results do not directly demonstrate structural transitions. Furthermore, the diffraction peak observed at approximately 2θ ≈ 28° may be associated with crystalline mineral components previously reported in cactus tissues [67]. Overall, diffraction profiles and crystalline characteristics varied across maturity stages within both the epidermis and cortex, with distinct diffraction patterns observed between the two tissues. These findings provide baseline information on the structural characteristics of cladodes and may support future investigations into their functional properties and potential applications.

## 4. Conclusions

The results revealed tissue-associated and developmental-stage-associated differences in the compositional and structural characteristics of edible cactus *Opuntia ficus-indica* (L.) Mill. cladodes, including variations in bioactive compounds such as total phenolic content, total flavonoid content, and organic acids. Structural analyses using FTIR and XRD further indicated distinct differences between tissues, which may be associated with their structural roles and physiological functions. Variations observed across maturity stages may also reflect changes in cell wall structure and biosynthetic processes during plant development. Overall, the results provide useful information on the compositional and structural characteristics of cladodes. Morphological and anatomical characterization further provides fundamental information on cladode structure, contributing to a better understanding of tissue characteristics across maturity stages. Together with the compositional analysis, these findings provide baseline information on the structural and chemical characteristics of edible cladodes for future research. The findings of this study provide baseline information on the compositional and structural characteristics of edible cactus cladodes within the scope of the present experimental design. Future studies employing complementary statistical approaches together with physiological and anatomical investigations would further strengthen the understanding of tissue-specific and maturity stage-related variations. Although edible cladodes may represent a promising plant-based resource, further studies on biological activity, bioavailability, functional properties, processing behavior, food formulation, safety, sensory properties, and digestibility are required before their potential applications in food products can be fully evaluated.

## Figures and Tables

**Table 1:** Description of cladode tissues of *O. ficus-indica* (L.) Mill.

Tissues	Appearance	Description
Epidermis (E)	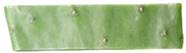	The outer green tissue of the cladode, enclosing the underlying succulent tissue. This tissue constitutes the photosynthetic surface and serves as the primary protective barrier against the external environment.
Cortex (C)	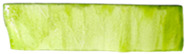	The succulent inner tissue located directly beneath the outer green tissue (epidermis), characterized by its water-rich parenchyma and occupying the internal region of the cladode.

The terms epidermis and cortex are used for descriptive purposes in this study to distinguish the outer green tissue and the inner succulent tissue of the cladode, and do not imply a formal anatomical classification.

**Table 2:** Bioactive compounds and antioxidant activity in cactus epidermis.

Sample	TPC (mg Gallic Acid/100 g DW)	TFC (mg Quercetin/100 g DW)	DPPH (mg L-Ascorbic Acid/100 g DW)	FRAP (mg FeSO_4_/100 g DW)
**1M**	273.49 ± 0.64 ^e^	918.06 ± 0.92 ^a^	0.89 ± 0.01 ^a^	1060.85 ± 1.21 ^e^
**3M**	289.77 ± 0.28 ^d^	615.76 ± 4.96 ^d^	0.48 ± 0.04 ^e^	1274.46 ± 1.45 ^b^
**6M**	407.79 ± 0.73 ^a^	743.96 ± 1.83 ^b^	0.62 ± 0.01 ^d^	1087.25 ± 5.51 ^d^
**9M**	305.05 ± 0.67 ^c^	489.80 ± 3.56 ^e^	0.74 ± 0.01 ^c^	1119.35 ± 1.31 ^c^
**12M**	350.35 ± 0.50 ^b^	697.82 ± 2.94 ^c^	0.79 ± 0.02 ^b^	1290.15 ± 1.47 ^a^

Values are expressed as mean ± SD of three technical (instrumental) measurements. Different lowercase letters within the same tissue indicate significant differences among maturity stages (*p* < 0.05). DW; dry weight.

**Table 3:** Bioactive compounds and antioxidant activity in cactus cortex.

Sample	TPC (mg Gallic Acid/100 g DW)	TFC (mg Quercetin/100 g DW)	DPPH (mg L-Ascorbic Acid/100 g DW)	FRAP (mg FeSO_4_/100 g DW)
**1M**	270.98 ± 1.09 ^b^	853.11 ± 3.17 ^a^	0.36 ± 0.02 ^c^	921.12 ± 1.87 ^a^
**3M**	279.27 ± 0.26 ^a^	450.48 ± 0.98 ^c^	0.48 ± 0.01 ^a^	892.76 ± 0.77 ^b^
**6M**	213.35 ± 1.03 ^c^	402.95 ± 1.11 ^d^	0.33 ± 0.01 ^c^	762.69 ± 3.06 ^c^
**9M**	164.92 ± 0.58 ^e^	644.70 ± 1.45 ^b^	0.48 ± 0.01 ^a^	580.43 ± 0.64 ^e^
**12M**	178.35 ± 1.01 ^d^	363.20 ± 4.01 ^e^	0.42 ± 0.03 ^b^	744.07 ± 3.39 ^d^

Values are expressed as mean ± SD of three technical (instrumental) measurements. Different lowercase letters within the same tissue indicate significant differences among maturity stages (*p* < 0.05). DW; dry weight.

**Table 4:** Phenolic acid and flavonoid content of cactus (epidermis).

Parameter	1M	3M	6M	9M	12M	LOD (ppm)	LOQ (ppm)	Recovery (%)
**Phenolic acid content (µg/g DW)**								
Caffeic acid	5.62 ± 0.21 ^b^	4.72 ± 0.10 ^c^	4.25 ± 0.09 ^d^	0.99 ± 0.09 ^e^	8.61 ± 0.01 ^a^	0.180	9.629	93–102
Chlorogenic acid	224.10 ± 1.20 ^a^	109.74 ± 0.33 ^c^	170.56 ± 0.85 ^b^	54.54 ± 0.25 ^d^	15.98 ± 0.68 ^e^	0.109	4.853	80–110
Cinnamic acid	2.12 ± 0.04 ^b^	3.08 ± 0.07 ^a^	2.13 ± 0.09 ^b^	1.25 ± 0.04 ^c^	2.15 ± 0.18 ^b^	0.025	1.437	98–102
Ferulic acid	5.04 ± 0.11 ^b^	8.95 ± 0.15 ^a^	5.00 ± 0.42 ^b^	1.15 ± 0.00 ^d^	4.20 ± 0.07 ^c^	0.069	13.926	98–103
Gallic acid	47.75 ± 1.68 ^cd^	57.97 ± 0.10 ^a^	55.42 ± 0.08 ^b^	46.90 ± 0.25 ^d^	48.46 ± 0.43 ^c^	0.106	19.667	98–102
Gentisic acid	668.23 ± 6.77 ^a^	503.00 ± 5.75 ^c^	498.46 ± 4.53 ^c^	384.58 ± 4.86 ^d^	544.71 ± 3.42 ^b^	0.043	2.531	98–102
*p*-coumaric acid	0.29 ± 0.00 ^b^	0.28 ± 0.04 ^b^	0.31 ± 0.02 ^b^	0.09 ± 0.01 ^c^	1.54 ± 0.04 ^a^	0.046	8.486	97–101
*p*-hydroxybenzoic acid	46.17 ± 0.94 ^b^	34.44 ± 0.56 ^e^	53.48 ± 0.22 ^a^	38.69 ± 0.45 ^d^	40.71 ± 0.09 ^c^	0.085	7.375	93–103
Protocatechuic acid	234.45 ± 2.17 ^a^	154.04 ± 1.68 ^e^	210.59 ± 2.32 ^b^	169.48 ± 0.61 ^d^	189.14 ± 0.84 ^c^	0.028	2.099	97–101
Sinapic acid	3.22 ± 0.13 ^d^	6.16 ± 0.04 ^b^	2.80 ± 0.05 ^e^	7.55 ± 0.02 ^a^	5.14 ± 0.09 ^c^	0.045	2.753	99–101
Syringic acid	4.66 ± 0.09 ^d^	9.82 ± 0.09 ^a^	3.99 ± 0.05 ^e^	7.16 ± 0.31 ^b^	6.56 ± 0.05 ^c^	0.050	13.516	95–103
Vanillic acid	14.18 ± 0.09 ^a^	14.45 ± 0.13 ^a^	14.10 ± 0.10 ^a^	4.04 ± 0.13 ^b^	3.91 ± 0.06 ^b^	0.091	6.344	94–101
***total***	1255.85 ± 3.39 ^a^	906.66 ± 7.14 ^c^	1021.09 ± 2.23 ^b^	716.43 ± 4.65 ^e^	871.11 ± 2.47 ^d^	0.091	6.344	94–101
**Flavonoid content (µg/g DW)**								
Apigenin	21.86 ± 0.93 ^c^	49.98 ± 1.5 ^a^	18.11 ± 0.06 ^d^	15.35 ± 0.57 ^e^	28.69 ± 0.30 ^b^	0.523	1.189	96–102
Kaempferol	18.40 ± 1.12 ^c^	26.24 ± 0.51 ^a^	19.82 ± 0.60 ^b^	10.43 ± 0.14 ^e^	15.09 ± 0.42 ^d^	7.854	24.453	98–105
Myricetin	111.19 ± 7.78 ^b^	87.34 ± 3.22 ^c^	87.46 ± 3.27 ^c^	47.55 ± 1.01 ^d^	129.36 ± 5.16 ^a^	7.617	15.388	97–110
Quercetin	27.75 ± 1.57 ^a^	19.13 ± 0.10 ^b^	18.28 ± 0.20 ^bc^	11.70 ± 0.19 ^d^	17.00 ± 0.60 ^c^	1.023	1.706	94–101
Rutin	49.15 ± 0.16 ^e^	96.25 ± 0.71 ^c^	156.74 ± 0.61 ^b^	70.99 ± 3.94 ^d^	264.25 ± 8.60 ^a^	0.723	9.744	99–101
***total***	228.35 ± 5.7 ^d^	278.94 ± 3.21 ^c^	300.41 ± 3.89 ^b^	156.03 ± 4.65 ^e^	454.39 ± 14.34 ^a^			

Values are expressed as mean ± SD of three technical (instrumental) measurements. Different lowercase letters within the same tissue indicate significant differences among maturity stages (*p* < 0.05). DW; dry weight.

**Table 5:** Phenolic acid and flavonoid content of cactus (cortex).

Parameter	1M	3M	6M	9M	12M	LOD (ppm)	LOQ (ppm)	Recovery (%)
**Phenolic acid content (µg/g DW)**								
Caffeic acid	2.47 ± 0.02 ^b^	0.93 ± 0.03 ^e^	1.44 ± 0.08 ^c^	1.31 ± 0.10 ^d^	2.93 ± 0.05 ^a^	0.180	9.629	93–102
Chlorogenic acid	89.01 ± 0.59 ^a^	27.25 ± 0.39 ^b^	25.71 ± 0.32 ^c^	10.92 ± 0.62 ^d^	11.68 ± 0.54 ^d^	0.109	4.853	80–110
Cinnamic acid	0.40 ± 0.01 ^c^	0.33 ± 0.01 ^d^	0.42 ± 0.00 ^c^	0.54 ± 0.02 ^b^	1.10 ± 0.06 ^a^	0.025	1.437	98–102
Ferulic acid	0.88 ± 0.02 ^b^	0.95 ± 0.06 ^b^	0.57 ± 0.02 ^d^	0.67 ± 0.01 ^c^	1.11 ± 0.06 ^a^	0.069	13.926	98–103
Gallic acid	50.80 ± 0.38 ^b^	51.51 ± 0.16 ^a^	46.38 ± 0.30 ^c^	46.83 ± 0.31 ^c^	45.50 ± 0.19 ^d^	0.106	19.667	98–102
Gentisic acid	426.69 ± 4.92 ^a^	203.59 ± 2.46 ^c^	191.11 ± 2.11 ^e^	196.84 ± 1.63 ^d^	247.49 ± 3.30 ^b^	0.043	2.531	98–102
*p*-coumaric acid	0.23 ± 0.01 ^b^	0.18 ± 0.01 ^c^	0.09 ± 0.01 ^e^	0.12 ± 0.01 ^d^	1.89 ± 0.16 ^a^	0.046	8.486	97–101
*p*-hydroxybenzoic acid	10.75 ± 0.06 ^d^	9.83 ± 0.18 ^e^	17.12 ± 0.11 ^a^	16.31 ± 0.19 ^b^	11.38 ± 0.14 ^c^	0.085	7.375	93–103
Protocatechuic acid	118.44 ± 1.01 ^a^	45.86 ± 0.26 ^c^	46.02 ± 0.71 ^c^	40.78 ± 0.19 ^d^	110.67 ± 0.41 ^b^	0.028	2.099	97–101
Sinapic acid	1.11 ± 0.02 ^a^	0.67 ± 0.04 ^d^	1.09 ± 0.01 ^b^	0.77 ± 0.03 ^c^	0.49 ± 0.02 ^e^	0.045	2.753	99–101
Syringic acid	7.58 ± 0.08 ^a^	3.65 ± 0.11 ^c^	3.50 ± 0.14 ^c^	1.31 ± 0.08 ^d^	4.82 ± 0.02 ^b^	0.050	13.516	95–103
Vanillic acid	12.65 ± 1.33 ^a^	6.38 ± 0.84 ^c^	7.71 ± 0.17 ^b^	5.71 ± 0.19 ^c^	3.01 ± 0.30 ^d^	0.091	6.344	94–101
*total*	721.00 ± 2.51 ^a^	351.14 ± 3.19 ^c^	341.16 ± 2.26 ^d^	322.10 ± 1.16 ^e^	442.06 ± 3.18 ^b^	0.091	6.344	94–101
**Flavonoid content (µg/g DW)**								
Apigenin	15.24 ± 1.12 ^b^	14.08 ± 1.05 ^bc^	13.37 ± 0.28 ^c^	16.82 ± 0.07 ^a^	9.65 ± 0.19 ^d^	0.523	1.189	96–102
Kaempferol	17.71 ± 1.17 ^b^	23.63 ± 0.36 ^a^	14.71 ± 0.36 ^d^	14.04 ± 0.23 ^e^	16.53 ± 0.27 ^c^	7.854	24.453	98–105
Myricetin	59.78 ± 3.17 ^a^	44.97 ± 0.70 ^b^	36.68 ± 1.68 ^d^	40.38 ± 1.09 ^c^	40.31 ± 0.39 ^c^	7.617	15.388	97–110
Quercetin	22.77 ± 1.93 ^b^	28.64 ± 1.71 ^a^	15.06 ± 0.32 ^d^	18.51 ± 0.39 ^c^	28.01 ± 0.69 ^a^	1.023	1.706	94–101
Rutin	2.43 ± 0.17 ^d^	3.57 ± 0.33 ^b^	4.43 ± 0.12 ^a^	3.30 ± 0.14 ^b^	2.96 ± 0.10 ^c^	0.723	9.744	99–101
*total*	117.94 ± 4.58 ^a^	114.89 ± 1.84 ^a^	84.26 ± 1.55 ^d^	93.05 ± 1.16 ^c^	97.46 ± 0.28 ^b^			

Values are expressed as mean ± SD of three technical (instrumental) measurements. Different lowercase letters within the same tissue indicate significant differences among maturity stages (*p* < 0.05). DW; dry weight.

**Table 6:** Organic acid content of cactus (epidermis).

Sample	Oxalic Acid (mg/g DW)	Malic Acid (mg/g DW)	Succinic Acid (mg/g DW)	Fumaric Acid (mg/g DW)
1M	15.29 ± 0.02 ^c^	10.74 ± 0.09 ^d^	11.62 ± 0.41 ^b^	20.46 ± 0.25 ^a^
3M	13.61 ± 0.04 ^e^	13.38 ± 0.42 ^c^	7.56 ± 0.02 ^c^	10.86 ± 0.13 ^b^
6M	16.27 ± 0.02 ^b^	13.61 ± 0.05 ^c^	12.31 ± 0.37 ^b^	0.59 ± 0.01 ^c^
9M	15.18 ± 0.02 ^d^	15.29 ± 0.01 ^b^	11.63 ± 0.43 ^b^	0.61 ± 0.01 ^c^
12M	47.20 ± 0.06 ^a^	16.27 ± 0.02 ^a^	14.47 ± 0.55 ^a^	0.51 ± 0.01 ^c^

Values are expressed as mean ± SD of three technical (instrumental) measurements. Different lowercase letters within the same tissue indicate significant differences among maturity stages (*p* < 0.05). DW; dry weight.

**Table 7:** Organic acid content of cactus (cortex).

Sample	Oxalic Acid (mg/g DW)	Malic Acid (mg/g DW)	Succinic Acid (mg/g DW)	Fumaric Acid (mg/g DW)
1M	8.19 ± 0.03 ^e^	9.67 ± 0.14 ^e^	2.76 ± 0.01 ^e^	34.41 ± 1.89 ^a^
3M	8.87 ± 0.16 ^d^	44.34 ± 0.3 ^d^	8.86 ± 0.16 ^d^	0.27 ± 0.01 ^b^
6M	10.78 ± 0.49 ^c^	45.37 ± 0.09 ^c^	10.89 ± 0.03 ^c^	0.33 ± 0.02 ^b^
9M	14.69 ± 0.29 ^b^	46.82 ± 0.04 ^b^	12.69 ± 0.69 ^b^	0.54 ± 0.01 ^b^
12M	46.82 ± 0.04 ^a^	52.26 ± 0.07 ^a^	14.72 ± 0.25 ^a^	0.53 ± 0.02 ^b^

Values are expressed as mean ± SD of three technical (instrumental) measurements. Different lowercase letters within the same tissue indicate significant differences among maturity stages (*p* < 0.05). DW; dry weight.

## Data Availability

The original contributions presented in this study are included in the article/Supplementary Material. Further inquiries can be directed to the corresponding author.

## Supplementary Materials

The following supporting information can be downloaded at: https://www.mdpi.com/article/10.3390/foods15142520/s1. Table S1: Calibration and validation parameters of the HPLC-PDA method for the determination of phenolic acids and flavonoids. Table S2: Calibration parameters for the HPLC determination of organic acids.
