# Supplementary material for: Characterization of Bioactive Composition and Structural Properties of Epidermis and Cortex Tissues in Edible Cactus [Opuntia ficus-indica (L.) Mill.] Cladodes at Different Maturity Stages
Source: Foods. 2026 Jul 16;15(14):2520. doi: 10.3390/foods15142520 (PMC13409659; doi:10.3390/foods15142520)
Supplement: Supplementary file 1 [file foods-15-02520-s001.zip › foods-4415327-supplementary.pdf]

Supplementary Table S1. Calibration and validation parameters of the HPLC-PDA method for the determination of phenolic acids and flavonoids.

Compound	Calibration range (µg/mL)	Regression equation	R^2	LOD (ppm)	LOQ (ppm)	Recovery (%)
**Phenolic acids**						
Gallic acid	6.25–100	y = 78,019x − 1,274.3	0.9998	0.106	19.667	98–102
Protocatechuic acid	6.25–100	y = 47,645x + 1,893	1.0000	0.028	2.099	97–101
p-Hydroxybenzoic acid	6.25–100	y = 78,883x − 73,853	1.0000	0.085	7.375	93–103
Vanillic acid	6.25–100	y = 49,696x + 2,158.4	0.9998	0.091	6.344	94–101
Caffeic acid	6.25–100	y = 156,014x − 8,841.6	0.9993	0.180	9.629	93–102
Chlorogenic acid	6.25–100	y = 38,055x − 15,157	0.9998	0.109	4.853	80–110
Syringic acid	6.25–100	y = 72,068x − 8,841.6	0.9999	0.050	13.516	95–103
p-Coumaric acid	6.25–100	y = 175,186x − 286.17	1.0000	0.046	8.486	97–101
Ferulic acid	6.25–100	y = 150,983x − 2,196.5	0.9999	0.069	13.926	98–103
Sinapic acid	6.25–100	y = 140,735x − 634.42	1.0000	0.045	2.753	99–101
Gentisic acid	6.25–100	y = 43,642x + 8,207.3	0.9989	0.043	2.531	98–102
Cinnamic acid	6.25–100	y = 103,334x − 645.4	0.9998	0.025	1.437	98–102
**Flavonoids**						
Rutin	6.25–100	y = 18,543x + 4,168.1	0.9998	0.723	9.744	99–101
Myricetin	6.25–100	y = 51,540x − 77,311	0.9988	7.617	15.388	97–110
Quercetin	6.25–100	y = 46,930x + 237,033	0.9997	1.0231	1.706	94–101
Kaempferol	6.25–100	y = 22,910x − 22,300	0.9987	7.854	24.453	98–105
Apigenin	6.25–100	y = 23,380x − 14,228	0.9998	0.523	1.189	96–102

Calibration curves were constructed using five concentration levels (6.25–100 µg/mL) of authentic standards. LOD and LOQ were automatically calculated using Shimadzu LabSolutions software (Shimadzu Corporation, Kyoto, Japan) based on the standard deviation of the response and the slope of the calibration

Supplementary Table S2. Calibration parameters for the HPLC determination of organic acids.

**Organic acid**	**Calibration range (μg/mL)**	**Regression equation**	**R^2**
Oxalic acid	6.25–100	y = 12,548x + 68,589	0.9995
Malic acid	6.25–100	y = 2,267.4x − 381.94	0.9998
Succinic acid	6.25–100	y = 1,566.7x − 25,047	0.9998
Fumaric acid	6.25–100	y = 36,852x − 35,713	0.9997

Calibration curves were established using five concentration levels (6.25–100 μg/mL) of authentic standards.
